# Placental amino acid transport may be regulated by maternal vitamin D and vitamin D-binding protein: results from the Southampton Women's Survey

**DOI:** 10.1017/S0007114515001178

**Published:** 2015-05-05

**Authors:** J. K. Cleal, P. E. Day, C. L. Simner, S. J. Barton, P. A. Mahon, H. M. Inskip, K. M. Godfrey, M. A. Hanson, C. Cooper, R. M. Lewis, N. C. Harvey, the SWS Study Group

**Affiliations:** 1 Faculty of Medicine, Institute of Developmental Sciences, University of Southampton, Tremona Road, SouthamptonSO16 6YD, UK; 2 MRC Lifecourse Epidemiology Unit, University of Southampton, Tremona Road, SouthamptonSO16 6YD, UK; 3 NIHR Southampton Biomedical Research Centre, University of Southampton and University Hospital Southampton NHS Foundation Trust, Tremona Road, SouthamptonSO16 6YD, UK; 4 NIHR Musculoskeletal Biomedical Research Unit, University of Oxford, Nuffield Orthopedic Centre, Headington, OxfordOX3 7HE, UK

**Keywords:** Vitamin D, Amino acid transporters, Placenta

## Abstract

Both maternal 25-hydroxyvitamin D (25(OH)D) concentrations during pregnancy and placental amino acid transporter gene expression have been associated with development of the offspring in terms of body composition and bone structure. Several amino acid transporter genes have vitamin D response elements in their promoters suggesting the possible linkage of these two mechanisms. We aimed to establish whether maternal 25(OH)D and vitamin D-binding protein (VDBP) levels relate to expression of placental amino acid transporters. RNA was extracted from 102 placental samples collected in the Southampton Women's Survey, and gene expression was analysed using quantitative real-time PCR. Gene expression data were normalised to the geometric mean of three housekeeping genes, and related to maternal factors and childhood body composition. Maternal serum 25(OH)D and VDBP levels were measured by radioimmunoassay. Maternal 25(OH)D and VDBP levels were positively associated with placental expression of specific genes involved in amino acid transport. Maternal 25(OH)D and VDBP concentrations were correlated with the expression of specific placental amino acid transporters, and thus may be involved in the regulation of amino acid transfer to the fetus. The positive correlation of VDBP levels and placental transporter expression suggests that delivery of vitamin D to the placenta may be important. This exploratory study identifies placental amino acid transporters which may be altered in response to modifiable maternal factors and provides a basis for further studies.

Vitamin D insufficiency is common in women of childbearing age and is associated with reduced foetal growth and poor postnatal health^(^ ^1^ ^,^ ^2^ ^)^. The biologically inactive 25-hydroxyvitamin D (25(OH)D) is used to monitor vitamin D status, as this is the major circulating form^(^ ^3^ ^)^. In the Southampton Women's Survey (SWS), a prospective longitudinal study of maternal nutrition and lifestyle before and during pregnancy, it was found that lower maternal 25(OH)D was associated with morphological changes in the foetal femur^(^ ^4^ ^)^, lower neonatal fat mass and greater fat mass and lower grip strength in childhood^(^ ^5^ ^,^ ^6^ ^)^. Reduced 25(OH)D during late pregnancy was also associated with reduced bone mineral content in children at 9 years of age in another Southampton cohort study^(^ ^2^ ^)^.

The mechanisms underlying these associations are not fully understood, but are likely to involve the placenta, the sole conduit for nutrients from mother to fetus. We previously reported that placental mRNA expression of the vitamin D sensitive Ca transporter plasma membrane Ca ATPase 3 *(PMCA3)* and the imprinted gene Pleckstrin homology-like domain family A member 2 *(PHLDA2)* is associated with offspring bone mass development and composition^(^ ^7^ ^,^ ^8^ ^)^. Other than Ca transport, a key element for foetal bone development is placental amino acid transport. Placental amino acid transfer is vital for foetal growth^(^ ^9^ ^)^, and animal studies suggest that decreased amino acid transport precedes foetal growth restriction^(^ ^10^ ^)^. Amino acid transfer to the fetus involves amino acid transport across the microvillous and basal membranes of the placental syncytiotrophoblast^(^ ^11^ ^)^ and potentially metabolic interconversion within the placenta^(^ ^12^ ^)^. Placental amino acid transfer is thought to be regulated by maternal nutritional and hormonal factors^(^ ^13^ ^–^ ^15^ ^)^.

There are three classes of amino acid transporter in the human placenta; accumulative transporters, amino acid exchangers^(^ ^16^ ^,^ ^17^ ^)^ and facilitated transporters^(^ ^18^ ^)^ (Fig. 1). Accumulative transporters mediate net uptake of specific amino acids across the microvillous membrane (e.g. SNAT), and also play roles on the basal membranes such as uptake of foetal glutamate for placental glutamine synthesis (e.g. EAAT)^(^ ^12^ ^)^. The exchangers including LAT, y^+^LAT and ASCT use the gradients built up by accumulative transporters to drive uptake and transfer of other amino acids including many essential amino acids^(^ ^18^ ^)^. The facilitated transporters TAT1, LAT3 and LAT4 are essential for net amino acid transport to the fetus, and their gene expression in human placenta is associated with measures of foetal growth^(^ ^18^ ^)^. The factors that regulate these changes in gene expression are not understood. However, as these and several other amino acid transporters have vitamin D response elements (VDRE) in their promoter regions, they could theoretically be regulated at the transcriptional level by maternal vitamin D. Specifically, the biologically active 1,25 dihydroxyvitamin D regulates transcription of specific genes by binding the vitamin D receptor and interacting with VDRE in their promoter regions^(^ ^19^ ^,^ ^20^ ^)^.

**Fig. 1:**
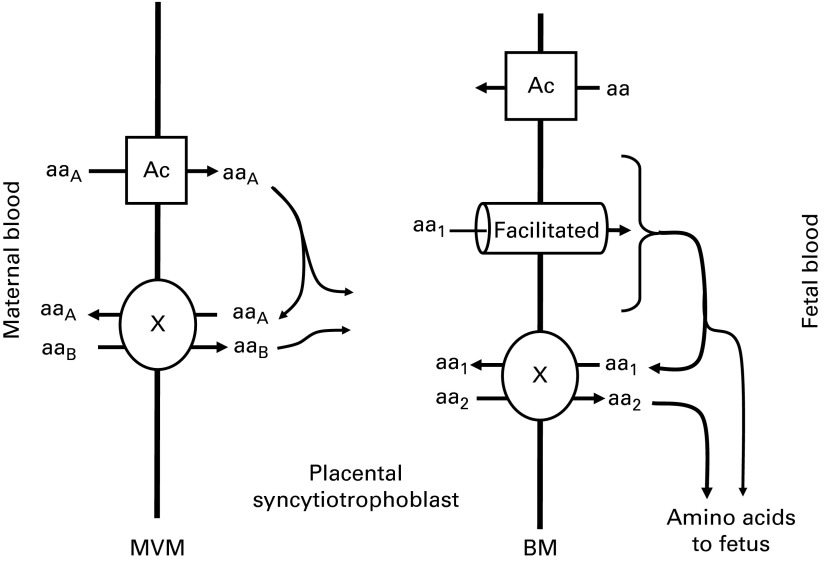
Transport of amino acids across the placental syncytiotrophoblast. Amino acids are transported across the microvillous membrane (MVM) into the placental syncytiotrophoblast by active accumulative transporters (Ac; e.g. SNAT) and exchangers (X; e.g. ASCT). Amino acids transported by accumulative transporters (aa_A_) are then exchanged back for those only transported by exchangers (aa_B_). Amino acids are transported out of the placenta across the basal membrane (BM) by facilitated transporters (TAT1, LAT3 and LAT4) and exchangers (X). The facilitated transporters transport specific amino acids (aa_1_) down their concentration gradient to the fetus. In order to transport other amino acids (aa_2_) to the fetus, aa_1_ must be exchanged for aa_2_ via exchangers (X).

We therefore investigated whether maternal 25(OH)D and vitamin D binding protein (VDBP) concentrations during pregnancy are related to gene expression of the amino acid transporters, essential for placental amino acid transfer. We used samples collected from a population based cohort, the SWS.

## Methods

The study was conducted according to the guidelines in the Declaration of Helsinki, and the Southampton and South West Hampshire Research Ethics Committee approved all procedures (276/97, 307/97, 089/99, 153/99, 005/03/t, 06/Q1702/104). Written informed consent was obtained from all participating women and by parents or guardians with parental responsibility on behalf of their children.

### Maternal measurements

We used data and samples from the SWS, a cohort study of 3158 pregnancies with information collected from the mothers before conception^(^ ^21^ ^)^. Non-pregnant women aged 20–34 years were recruited via their general practitioners; assessments of lifestyle, diet and anthropometry were performed by trained research nurses at study entry and then in early (11 weeks) and late (34 weeks) gestation among those women who became pregnant. Subscapular skinfold thicknesses were measured to the nearest 0·1 mm in triplicate using Harpenden skinfold callipers (Baty International)^(^ ^22^ ^)^.

At 34 weeks of gestation, a maternal venous blood sample was obtained and an aliquot of maternal serum was frozen at − 80°C. Serum 25(OH)D and VDBP concentrations were analysed by RIA (DiaSorin). The 25(OH)D assay measures both 25-hydroxyvitamin D2 and 25-hydroxyvitamin D3. The detection range for this 25(OH)D assay is 3·8–250 nmol/l. The assays met the requirements of the UK National Vitamin D External Quality Assurance Scheme, and intra- and inter-assay CV were < 10 %.

### Placental samples

Placentas were collected from term pregnancies within 30 min of delivery, and no clinical conditions such as pre-eclampsia or gestational diabetes. Placental weight was measured after removing blood clots, cutting the umbilical cord flush with its insertion into the placenta, trimming away surrounding membranes and removing the amnion from the basal plate. To ensure that the samples collected were representative of the placentas as a whole, five villous tissue samples were selected using a stratified random sampling method, and stored at − 80°C. For the present study, a cohort of 102 placentas was selected from 300 collected in total, based on availability of neonatal dual-energy X-ray absorptiometry (DXA) data.

### RNA extraction and complementary DNA synthesis

For each placenta five snap frozen samples were pooled and powdered in a frozen tissue press. Total RNA was extracted from 30 mg powdered placental tissue using the RNeasy fibrous tissue RNA isolation mini kit (Qiagen) according to the manufacturer's instructions. The integrity of total RNA was confirmed by agarose gel electrophoresis.

Total RNA (0·2 μg) was reverse transcribed with 0·5 μg random hexamer primer, 200 units Moloney murine leukaemia virus reverse transcriptase, 25 units recombinant RNasin ribonuclease inhibitor and 0·5 mm each of deoxyadenosine triphosphate, deoxycytidine triphosphate, deoxyguanosine triphosphate and deoxythymidine triphosphate in a final reaction volume of 25 μl in 1 ×  Moloney murine leukaemia virus reaction buffer (Promega). All 102 samples were produced in one batch to reduce variation.

### Probe and primer design

Intron spanning oligonucleotide probes and primers were designed using the Roche ProbeFinder version 2.45 for human. Probes were supplied by Roche from the human universal probe library and primers were synthesised by Eurogentec. Control genes were selected using the geNorm™ human Housekeeping Gene Selection Kit (Primer Design Limited).

### Target genes

The genes measured in the present study along with primer and probe details are listed in Table 1. And mRNA levels were measured using quantitative real-time PCR using a Roche LightCycler 480. For Roche universal probe library probes the cycle parameters were 95°C for 10 min, followed by forty cycles of 95°C for 15 s and 60°C for 1 min. For the primer design Perfect Probes, the cycle parameters were 95°C for 10 min, followed by forty cycles of 95°C for 10 s and 60 and 72°C for 15 s. Intra-assay CV's for each gene were 5–8 %. Each of the 102 samples was run on the same plate in triplicate. All mRNA levels are presented relative to the geometric mean of the three control genes, tyrosine 3-monooxygenase/tryptophan 5-monooxygenase activation protein, zeta polypeptide (*YWHAZ*), ubiquitin C (*UBC*) and topoisomerase (*TOP1*)^(^ ^23^ ^)^.

**Table 1:** Information on genes, primers and probes

Transporter	Gene	Gene ID	Genebank accession no.	Primers	Roche universal probe library no.
*ASCT1*	*SLC1A4*	6509	NM_003038.2	F: 5′-tttgcgacagcatttgctac-3′	78
				R: 5′-gcacttcatcatagagggaagg-3′	
*ASCT2*	*SLC1A5*	6510	NM_005628.2	F: 5′-gaggaatatcaccggaacca-3′	43
			NM_001145144.1	R: 5′-aggatgttcatcccctcca-3′	
*EAAT1*	*SLC1A3*	6507	NM_004172.4	F: 5′-ttgaactgaacttcggacaaatta-3′	76
				R: 5′-attccagctgccccaatact-3′	
*EAAT2*	*SLC1A2*	6506	NM_004171.3	F: 5′-aaaatgctcattctccctctaatc-3′	78
				R: 5′-gccactagccttagcatcca-3′	
*EAAT3*	*SLC1A1*	6505	NM_004170.4	F: 5′-agttgaatgacctggacttgg-3′	9
				R: 5′-gcagatgtggccgtgatac-3′	
*EAAT4*	*SLC1A6*	6511	NM_005071.1	F: 5′-tgcagatgctggtgttacct-3′	19
				R: 5′-gttgtccagggatgccata-3′	
*EAAT5*	*SLC1A7*	6512	NM_006671.4	F: 5′-cgcccaggtcaacaactac-3′	9
				R: 5′-gctgcagtggctgtgatact-5′	
*LAT1*	*SLC7A5*	8140	NM_003486.5	F: 5′-gtggaaaaacaagcccaagt-3′	25
				R: 5′-gcatgagcttctgacacagg-3′	
*LAT2*	*SLC7A8*	23428	NM_182728.1	F: 5′-ttgccaatgtcgcttatgtc-3′	17
			NM_012244.2	R: 5′-ggagcttctctccaaaagtcac-3′	
*LAT3*	*SLC43A1*	8501	NM_003627.5	F: 5′-gccctcatgattggctctta-3′	29
			NM_001198810.1	R: 5′-ccggcatcgtagatcagc-3′	
*LAT4*	*SLC42A2*	124935	NM_001284498.1	F: 5′-acaagtatggcccgaggaa-3′	3
			NM_152346.2	R: 5′-gcaatcagcaagcaggaaa-3′	
*SNAT1*	*SLC38A1*	81539	NM_030674.3	F: 5′-attttgggactcgcctttg-3′	47
			NM_001077484.1	R: 5′-agcaatgtcactgaagtcaaaagt-3′	
*SNAT2*	*SLC38A2*	54407	NM_018976.3	F: 5′-cctatgaaatctgtacaaaagattgg-3′	9
				R: 5′-ttgtgtacccaatccaaaacaa-3′	
*SNAT4*	*SLC38A4*	55089	NM_018018.4	F: 5′-tgttctggtcatccttgtgc-3′	29
			NM_001143824.1	R: 5′-aaaactgctggaagaataaaaatcag-3′	
*TAT1*	*SLC16A10*	117247	NM_018593.4	F: 5′-ggtgtgaagaaggtttatctacagg-3′	6
				R: 5′-agggccccaaagatgcta-3′	
*y^+^LAT1*	*SLC7A7*	9056	NM_001126105.1	F: 5′-acactgccgtgagaacctg-3′	72
			NM_001126106.1	R: 5′-aggagaggaaacccttcacc-3′	
*y^+^LAT2*	*SLC7A6*	9057	NM_001076785.1	F: 5′-gctgtgatcccccatacct-3′	66
			NM_003983.4	R: 5′-ggcacagttcacaaatgtcag-3′	
*4F2HC*	*SLC3A2*	6520	NM_001012661.1	F: 5′-tggttctccactcaggttga-3′	49
				R: 5′-cagccaaaactccagagcat-3′	

SLC, solute carrier; F, forward; R, reverse; *4F2HC*, type-II membrane glycoprotein heavy chain.

### Postnatal measurements

At birth (*n* 102) and 4 years of age (*n* 42–46) a whole-body DXA scan was obtained using a Hologic Discovery instrument (Hologic, Inc.) in paediatric scan mode (Apex 3.1 software), yielding fat mass, lean mass and bone mineral content. The CV for body composition analysis with the DXA instrument was 1·4–1·9 %.

### Statistics

Maternal and placental mRNA data that were not normally distributed were transformed logarithmically. Previous data showed that gene expression of the control genes and many of the target genes was higher in male than in female placentas^(^ ^24^ ^)^. Adjustment was therefore made for sex in the correlation analysis between mRNA and all other variables. Pearson's correlation coefficient (*r*_p_) was used to determine partial correlations adjusted for sex and gestational age between placental mRNA levels, neonatal body composition and maternal factors (IBM SPSS Statistics 20). The partial correlation between placental gene expression and maternal vitamin D measures was also adjusted for potential confounding factors: maternal sum of skinfold thickness, walking speed, parity and smoking during pregnancy. A value of *P*< 0·05 was accepted as statistically significant, and, given the observational nature of the study together with the substantial co-linearity among both predictors and outcomes, testing for multiple comparisons was felt to be inappropriate^(^ ^25^ ^)^.

## Results

### Characterisation of the subjects from the Southampton Women's Survey cohort

The mean age of the 102 mothers at the birth of their children was 30·9 (sd 3·9) years; 37·9 % were primiparous. 97 % of the women were of white European ethnicity. The median gestational age was 39·6 (inter-quartile range 38·8–40·7) weeks. The mean placental/foetal weight ratio was 0·13 (sd 0·02). Of the 102 placentas from SWS pregnancies studied here, fifty-three of the infants were male, forty-nine were female. The mean birth weight for males was 3547 (sd 417) g with 95 % between the 33rd and 51st centile based on UK growth charts. The mean birth weight for females was 3455 (sd 489) g with 95 % between the 36th and 59th centile.

### Maternal plasma vitamin D and placental gene expression

The 34-week plasma 25(OH)D levels were measured for ninety-one of the 102 women and VDBP levels for eighty-five of the 102 women. The mean 25(OH)D levels were 71·7 (sd 32·1) nmol/l with a range of 20–158 nmol/l. The mean VDBP levels were 5622 (sd 806) mg/l with a range of 4160–8570 mg/l. Of the women, 28·6 % were taking vitamin D supplements of 10 μg/d (400 IU/d). The mean vitamin D intake (from FFQ and data on supplements) from the ninety-eight available (out of 102) women's diets is 3·5 μg/d (ranging from 1·3 to 9·0 μg/d).

Of the genes investigated mRNA for *EAAT1*, *EAAT4* and *EAAT5* were not detected in human placenta.

**Fig. 2:**
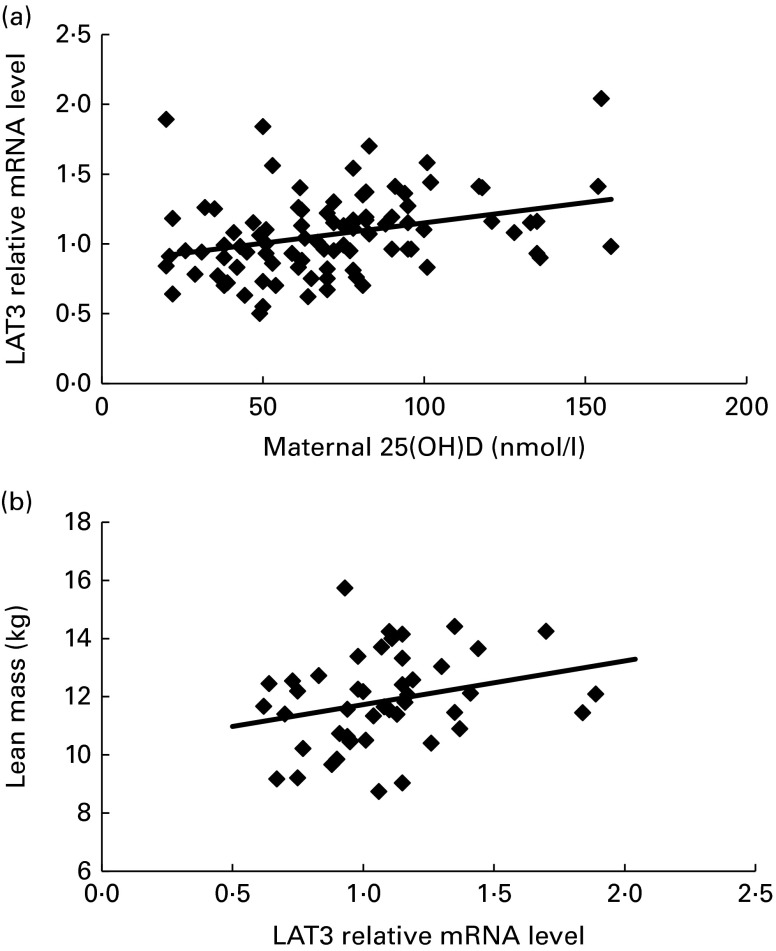
*LAT3* mRNA expression is associated with postnatal body composition. *LAT3* relative mRNA expression in human placenta is positively correlated with maternal 25-hydroxyvitamin D (25(OH)D) (*r*_p_ 0·31, *P*= 0·003, *n* 102) (a) and lean mass at 4 years of age (*r*_p_ 0·38, *P*= 0·01, *n* 46) (b).

In this subset of SWS woman, there was a positive correlation between maternal 34-week plasma 25(OH)D levels and the mRNA expression of *LAT3* (Fig. 2), *ASCT1* and *y* ^+^ *LAT1*, and a negative correlation with *SNAT1* (Table 2). Maternal VDBP levels correlated positively with mRNA expression of *TAT1*, *LAT3*, *LAT4*, *SNAT1*, *SNAT2*, *y* ^+^ *LAT2*, type-II membrane glycoprotein heavy chain *(4F2HC)*, and *EAAT3*, and there was a trend with *LAT1* (Table 2).

**Table 2:** The associations between placental amino acid transporter mRNA expression and maternal serum 25-hydroxyvitamin D and vitamin D binding protein levels

	34-week vitamin D (nmol/l)	Vitamin D binding protein (mg/l)	34-week vitamin D (nmol/l)	Vitamin D binding protein (mg/l)
	*r*	*P*	*r*	*P*	*r*	*P*	*r*	*P*
*TAT1*	0·07	0·50	0·23*	0·03*	0·14	0·21	0·12*	0·10*
*LAT3*	0·31*	0·003*	0·22*	0·04*	0·37*	0·003*	0·22*	0·05*
*LAT4*	− 0·12	0·25	0·28*	0·01*	− 0·13	0·26	0·28*	0·01*
*SNAT1*	− 0·23*	0·03*	0·25*	0·02*	− 0·20*	0·07*	0·23*	0·05*
*SNAT2*	0·01	0·96	0·23*	0·03*	0·04	0·70	0·23*	0·04*
*SNAT4*	0·14	0·19	0·08	0·45	0·12	0·30	0·12	0·29
*ASCT1*	0·23*	0·03*	0·06	0·62	0·20*	0·07*	0·11	0·33
*ASCT2*	0·04	0·74	0·18	0·10	0·05	0·63	0·17	0·14
*y^+^LAT1*	0·31*	0·003*	0·03	0·81	0·36*	0·001*	0·02	0·99
*y^+^LAT2*	0·04	0·73	0·26*	0·02*	− 0·08	0·94	0·33*	0·003*
*EAAT2*	0·12	0·26	− 0·07	0·53	0·06	0·56	− 0·01	0·91
*EAAT3*	0·09	0·39	0·30*	0·01*	0·12	0·24	0·29*	0·009*
*LAT1*	− 0·14	0·19	0·21*	0·06*	− 0·17	0·12	0·23*	0·04*
*LAT2*	− 0·08	0·44	0·18	0·10	− 0·07	0·54	0·17	0·14
*4F2HC*	− 0·12	0·26	0·25*	0·02*	− 0·08	0·48	0·23*	0·04*
	Adjusted for sex and dGA	Adjusted for sex, dGA and maternal confounding factors

*4F2HC*, type-II membrane glycoprotein heavy chain; dGa, days gestational age.

*P< 0·05.

When the correlation was also adjusted for maternal confounding factors (maternal sum of skinfold thickness, walking speed, parity and smoking during pregnancy) all correlations were still present, except for the relationships between 25(OH)D and *ASCT1*, and VDBP and *TAT1*, which were no longer statistically significant at the *P*< 0·05 level (Table 2). The adjusted data also showed a positive association between VDBP and *LAT1* mRNA (Table 2).

### Neonatal body composition

At birth, there were no significant associations between placental amino acid transporter gene expression and neonatal lean mass, fat mass, or bone mineral content (data not shown).

At 4 years of age total lean mass was positively associated with *LAT3* (Fig. 2), *y* ^+^ *LAT1* and *TAT1* mRNA expression (Table 3). Bone mineral density was positively associated with *LAT4* mRNA and negatively associated with *ASCT2* and *EAAT3* mRNA expression (Table 3). *EAAT3* mRNA expression levels (*n* 42) were also negatively associated with bone mineral content (*r*_p_ − 0·46, *P*= 0·003) and total bone area (cm^2^ without heads; *r*_p_ − 0·43, *P*= 0·01). *SNAT4* (*r*_p_ − 0·40, *P*= 0·01) and *y* ^+^ *LAT2* (*r*_p_ − 0·32, *P*= 0·04) expression levels were negatively associated with total bone area.

**Table 3:** The associations between placental amino acid transporter mRNA expression and 4-year-old dual-energy X-ray absorptiometry (DXA) measurements of body composition

	Total lean (kg) (*n* 46)	Total Prentice BMD (g), without heads (*n* 42)
4 year DXA	*r*	*P*	*r*	*P*
*TAT1*	0·33*	0·03*	− 0·17	0·28
*LAT3*	0·38*	0·01*	− 0·15	0·33
*LAT4*	− 0·09	0·57	0·41*	0·01*
*SNAT1*	− 0·12	0·45	0·06	0·72
*SNAT2*	0·06	0·68	− 0·11	0·49
*SNAT4*	0·08	0·62	− 0·18	0·26
*ASCT1*	0·23	0·13	− 0·27	0·09
*ASCT2*	0·24	0·11	− 0·42*	0·01*
*y^+^LAT1*	0·31*	0·04*	− 0·25	0·11
*y^+^LAT2*	0·20	0·18	− 0·27	0·09
*EAAT2*	0·28	0·07	0·04	0·82
*EAAT3*	− 0·04	0·80	− 0·59*	0·00 005*
*LAT1*	− 0·21	0·16	0·09	0·59
*LAT2*	− 0·04	0·82	− 0·02	0·90
*4F2HC*	0·05	0·73	0·14	0·38

BMD, bone mineral density; *4F2HC*, type-II membrane glycoprotein heavy chain.

*P< 0·05.

## Discussion

Many genes related to placental function may be regulated directly or indirectly by vitamin D. The present study aimed to establish whether there are relationships between maternal vitamin D levels and changes in gene expression in placentas from the SWS. Maternal 25(OH)D and VDBP levels were positively associated with placental expression of genes involved in amino acid transport. This suggests that maternal vitamin D status may regulate the expression of placental amino acid transporters, and potentially influence the transfer of amino acids to the fetus and subsequent foetal growth. The observations that VDBP was associated with the expression of twice as many genes as vitamin D suggests that delivery of vitamin D to the placenta may be a crucial determinant of vitamin D activity. The associations seen may however, involve a more complex relationship between maternal vitamin D status and maternal body composition.

### Vitamin D

Placental amino acid transport is important for foetal growth and development, so understanding how the amino acid transporters are regulated in the placenta will help us understand the mechanisms underlying foetal growth restriction and the associated postnatal phenotype. Maternal vitamin D status has also been shown to associate with both foetal and neonatal growth, and, taken with the fact that it modulates gene transcription; this suggests there may be an interaction between vitamin D and placental amino acid transport. This interaction could be a direct effect of vitamin D, and its receptor acting directly on the placental amino acid transporter genes at a VDRE or an indirect effect mediated via vitamin D's activation of another gene. Both the *LAT3* and *ASCT1* genes have been shown to have VDRE in their promoter region^(^ ^26^ ^)^, which could underlie the association between their mRNA expression and maternal 25(OH)D levels. Vitamin D can also down-regulate gene expression via vitamin D receptor, blocking the activity of the cyclic AMP response element in the promoter^(^ ^27^ ^)^. This may explain the observed negative association between 25(OH)D and *SNAT1* mRNA expression, a gene regulated by cyclic AMP at the cyclic AMP response element^(^ ^28^ ^)^. Vitamin D can also directly affect gene transcription by an interaction between vitamin D receptor and histone acetyltransferases, leading to an open/active chromatin state^(^ ^29^ ^)^. The amino acid transporter genes could therefore be in a region of DNA, affected by vitamin D-mediated epigenetic changes, or could be regulated indirectly via an effect on another gene in the placenta.

The relationship between vitamin D and placental function may be more complex than vitamin D receptor-mediated changes in placental gene expression, and could be very indirect via an effect on maternal physiology or metabolism. It could be that vitamin D levels are influencing aspects of the maternal environment, which in turn regulate placental gene expression. Alternatively, maternal factors could simply be regulating both vitamin D levels and placental amino acid transporter expression in a similar manner. Plasma vitamin D status is known to be related to factors such as maternal smoking, parity and BMI^(^ ^30^ ^)^. It could be that maternal body composition is influencing the placenta, as a signal reflecting the mother's nutrient reserves and capacity to support the pregnancy. We have previously demonstrated an association between maternal muscle mass and placental amino acid transfer, indicating that maternal body composition can affect placental amino acid handling^(^ ^31^ ^)^.

Vitamin D levels could therefore be a proxy for another aspect of the maternal environment, and not a direct mediator of amino acid transporter expression levels. When we corrected our correlation analysis to adjust for maternal factors, we did indeed see that the amino acid transporters *ASCT1* and *SNAT1* were no longer related to the maternal 25(OH)D levels. These transporters may therefore be regulated by aspects of maternal body composition rather than vitamin D status, or vitamin D levels may be mediating the effects of body composition on the placenta. *LAT3* and *y* ^+^ *LAT1* did still show strong associations with maternal 25(OH)D levels, suggesting that it is the vitamin D rather than body composition that affects their regulation. Further studies are needed to establish the mechanisms underlying this association.

Interestingly, there were a number of positive associations between VDBP and amino acid transporter expression levels. This suggests that the delivery of the vitamin D to the placenta by its binding protein may be an important determinant of vitamin D action, possibly mediated by receptor-mediated endocytosis^(^ ^32^ ^)^. Further investigation into the uptake of vitamin D and levels of the active 1,25 dihydroxyvitamin D within the placenta is needed. This will help us understand and improve the effects of 25(OH)D supplementation during pregnancy, which may also require the VDBP to be upregulated.

### Postnatal outcome

We previously reported that placental *TAT1* and *LAT3* mRNA expression levels in this cohort are positively related to measures of foetal growth, with *TAT1* mRNA being associated with foetal growth in terms of lean mass^(^ ^18^ ^)^. Consistent with these observations we found that *y* ^+^ *LAT1*, *TAT1* and *LAT3* mRNA expression in placentas are positively related to 4-year-old lean mass. As lean mass contains a high proportion of muscle, a protein-rich tissue, its growth will require a substantial amino acid supply, and so it may rely on appropriate amino acid supply in early development.

### Limitations

The present study has the advantage of using a well characterised population representative of the general population, with detailed phenotyping of mother–offspring pairs. The placentas and offspring included in this study were of the mothers who allowed DXA measurements to be undertaken. The women whose offspring had DXA measures, compared to those that did not, were slightly older and tended to be better educated. They do represent a wide range of maternal age and family backgrounds, and all comparisons were internal to the selected subset. When comparing the vitamin D levels in the women with placental samples *v*. the whole cohort they look very similar with a slightly higher mean, but a similar standard deviation; 71·7 (sd 32·1) nmol/l, *n* 91 *v*. 64·2 (sd 30·9) nmol/l, *n* 2178. In the present study we were only able to measure the inactive 25(OH)D, which is thought to be the best measure of vitamin D status. Further studies would be enhanced by measuring the level of active 1,25 dihydroxyvitamin D within the placental tissue, and relating this directly to gene expression. The exploratory nature of the present study, small sample size and the possibility of chance findings need to be acknowledged. In particular, we had reduced numbers at 4 years of age (42–46 mother–offspring pairs) due to participants' not returning for measurement. The measures made in this sub-set at 4 years of age were representative of the whole cohort, for example the mean lean mass was 11·8 (sd 1·6) kg, *n* 46 compared to 12·0 (sd 1·5) kg, *n* 743. These numbers did, however, give us greater than 90 % power to detect a correlation coefficient of 0·5. Compared to adults, DXA assessment of body composition in children is more problematic due to their smaller size and tendency to move. These DXA measures were, however, validated previously in piglets using biochemical assessment of carcass N content and lipid extraction to determine lean and fat mass, respectively^(^ ^33^ ^)^. In the present study specific paediatric software was used, and movement artefacts were minimal. While the present study focused on the actions of vitamin (as a transcription factor) on the expression of key placental genes, it would also have been interesting to study the effect of a wider range of factors including maternal and foetal amino acid levels. It is important to remember that the regulation of gene function and physiology are complex and will rarely be dependent on a single factor. It is not possible in this observational study to determine whether the observed associations are causal. Nevertheless, the patterns of observations are indicative of a role for vitamin D in the regulation of placental amino acid transporter expression, and it forms, we think, the basis for future studies.

### Conclusion

In conclusion the present study demonstrates relationships between maternal vitamin D levels, and in particular VDBP and placental gene expression. As there are associations between vitamin D and body composition, these observations provide a possible mechanism by which maternal factors influence placental function. Further work needs to be undertaken to investigate the association between maternal VDBP and placental gene expression, and whether these are direct or indirect effects.
